# Suspension-Sprayed Calcium Phosphate Coatings with Antibacterial Properties

**DOI:** 10.3390/jfb15100281

**Published:** 2024-09-25

**Authors:** Maria Carolina Lanzino, Long-Quan R. V. Le, Anika Höppel, Andreas Killinger, Wolfgang Rheinheimer, Sofia Dembski, Ali Al-Ahmad, Hermann O. Mayr, Michael Seidenstuecker

**Affiliations:** 1 Institute for Manufacturing Technologies of Ceramic Components and Composites (IFKB), University of Stuttgart, 70569 Stuttgart, Germany; andreas.killinger@ifkb.uni-stuttgart.de (A.K.); wolfgang.rheinheimer@ifkb.uni-stuttgart.de (W.R.); 2 G.E.R.N. Center of Tissue Replacement, Regeneration & Neogenesis, Department of Orthopedics and Trauma Surgery, Faculty of Medicine, Albert-Ludwigs-University of Freiburg, Hugstetter Straße 55, 79106 Freiburg, Germany; long-quan.le@uniklinik-freiburg.de (L.-Q.R.V.L.); hermann.mayr@uniklinik-freiburg.de (H.O.M.); michael.seidenstuecker@uniklinik-freiburg.de (M.S.); 3 Department of Tissue Engineering and Regenerative Medicine (TERM), University Hospital Würzburg, 97070 Würzburg, Germany; anika.hoeppel@isc-extern.fraunhofer.de (A.H.); sofia.dembski@isc.fraunhofer.de (S.D.); 4 Fraunhofer Institute for Silicate Research ISC, 97082 Würzburg, Germany; 5 Department of Operative Dentistry and Periodontology, Center for Dental Medicine, Faculty of Medicine, Albert-Ludwigs-University of Freiburg, 79106 Freiburg, Germany; ali.al-ahmad@uniklinik-freiburg.de

**Keywords:** CaP, supraparticles, joint, coating, antimicrobial activity

## Abstract

Prosthesis loosening due to lack of osteointegration between an implant and surrounding bone tissue is one of the most common causes of implant failure. Further, bacterial contamination and biofilm formation onto implants represent a serious complication after surgery. The enhancement of osteointegration can be achieved by using bioconductive materials that promote biological responses in the body, stimulating bone growth and thus bonding to tissue. Through the incorporation of antibacterial substances in bioconductive, biodegradable calcium phosphate (CaP) coatings, faster osteointegration and bactericidal properties can be achieved. In this study, Cu-doped CaP supraparticles are spray-dried and suspension-sprayed CaP ceramic coatings with antibacterial properties are prepared using high-velocity suspension flame spraying (HVSFS). The objective was to increase the coatings’ porosity and investigate which Cu-doped supraparticles have the strongest antibacterial properties when introduced into the coating layers. Biocompatibility was tested on human Osteosarcoma cells MG63. A porosity of at least 13% was achieved and the supraparticles could be implemented, enhancing it up to 16%. The results showed that the addition of Cu-doped supraparticles did not significantly reduce the number of viable cells compared to the Cu-free sample, demonstrating good biocompatibility. The antimicrobial activity was assessed against the bacterial strains *Escherichia coli* and *Staphylococcus aureus*, with Safe Airborne Antibacterial testing showing a significant reduction in both Gram-positive and Gram-negative strains on the Cu-doped coatings.

## 1. Introduction

Implant failure after arthroplasty, a common challenge in the medical field, often results from poor bonding between the implant and the surrounding bone, alongside issues like bacterial contamination and biofilm formation [1,2,3,4,5,6]. Effectively addressing these problems requires a comprehensive strategy that combines efforts to improve implant integration with the bone and provides strong protection against bacteria. Recent scientific studies have spotlighted the use of bioconductive materials to enhance the integration of implants with surrounding bone tissue [3,7]. The concept of bioconductive materials was firstly introduced by Larry Hench in the late 1960s, when he researched the capability of certain glasses to bonding to living bone [8,9]. Bioconductive materials trigger a biological response and induce hydroxyapatite (HAp) precipitation in contact with body fluid at the implant–tissue interface, promoting bone growth and ensuring better adherence to the tissue (bioconductive fixation) [10]. In more recent years, other bioconductive ceramic materials have been discovered and investigated [11]. In particular, the coating of biomaterials with CaP coatings has become a well-researched topic for medical applications [12]. Osteoconductive CaP coatings promote bone healing and lead to the faster integration and fixation of the implant into the bone, reducing the risk for an early loosening of the artificial joints [13]. The mechanism is based on the previously described dissolution and precipitation process that occurs when CaP enters the body, resulting in the formation of bone-like apatite crystals on the surface of implants [14].

A plasma-sprayed HAp coating has become state-of-the-art for orthopedic implants [15,16]. Tricalcium phosphate (TCP) is known to be resorbed by the body faster than HAp [17,18]. The chemical dissolution in simulated body fluid (SBF) is inversely proportional to the Ca/P molar ratio, which explains this property, since HAp has a Ca/P ratio of 1.67 while TCP shows a ratio of 1.5 [14,19]. When applying thin coating layers on the surface of an implant, the speed of the degradation of the CaP coating and the bone regeneration kinematics play a key role. When using resorbable ceramics, these should be synchronized. While the coating degrades and is absorbed by the body, it should be replaced by the new bone tissues. In this way, secondary stability can be achieved faster [20].

Adjusting the porosity, as highlighted already in 1995 by Cao et al., becomes a key aspect of our study because it helps cells to penetrate the coating, replacing the coating and thus promoting superior bone growth and sufficient stability at the interface [10]. Additionally, through the pores, the faster degradation of the coating layers might also be achieved, speeding up the dynamic processes at the ceramic–bone interfaces after implantation and thus ensuring stability. The degradation properties make CaP materials also suitable as carrier for antibacterial agents. Through their dissolution, a gradual release of the antibacterial substances such as metal ions can be assured [20,21,22,23,24].

The coating of joint implants with CaP can be achieved using various techniques, each offering distinct advantages and limitations. Common methods include thermal spraying, electrochemical deposition and sol–gel processes. Other methods include various gas-phase deposition processes, but these have not yet played a role in commercial use [25,26,27,28]. An alternative approach for coating CaP onto metal substrates is micro-arc oxidation (MAO). This allows the adjustments in both the thickness and porosity of the coating [29].

Among the available methods, thermal spraying stands out for its efficiency, as it enables the application of coatings in a single process step, typically completed within a few minutes. This characteristic is particularly beneficial for high-volume production. For decades, plasma spraying has been the most prevalent and widely used technique for coating non-cementable joint implants, which are anchored in bone via the so called press-fit mechanism [25,30].

In this study, the coatings were applied by high-velocity suspension flame spraying (HVSFS), a technique which was developed at the University of Stuttgart [31]. The HVSFS process gives the possibility to spray particles with significantly higher kinetic energy than APS [32]. Furthermore, the use of suspensions instead of powders allows one to utilize submicron- and nanopowders. This leads to the possibility of producing finely structured and thin coatings that can be optimized for a specific application [33,34].

It has already been shown that HVSFS can be used to produce layers of bioconductive materials and to dope them with antimicrobial metal ions to prevent biofilm formation [7,35,36]. In those studies, a full resorption of the coatings was not possible because of the low porosity.

Based on these findings, this study, being a step of the project “Thin degradable coatings for the optimization of osteointegration associated with simultaneous infection prophylaxis”, funded by the German Research Foundation (DFG), focuses on developing thin, bioresorbable and porous CaP coatings with antibacterial properties, applied using HVSFS, a crucial step toward speeding up implant integration and strengthening the ability of implants to fight bacteria. A thickness of 30 µm and a porosity of at least 9–10% are set as targets. These parameters play a crucial role in facilitating the faster resorption of the coating by the body. The underlying concept, as mentioned before, is that new bone tissue grows onto the coating, gradually replacing it as the coating itself degrades over time. These specific values were chosen to optimize the balance between structural integrity and the rate of resorption. Furthermore, producing such a thin coating is not feasible with the current state-of-the-art process, APS, highlighting the need for alternative methods. Thus, the porosity of the coatings became the focus of this research, since in past studies, very dense coatings were achieved. We pursued two ways to improve it. One way was to increase it by adjusting the coating parameters; the other was to introduce supraparticles into the coating to create pores between the matrix layers [36]. The supraparticles were doped with Cu in order to achieve antibacterial properties [37]. In this manner, the Cu incorporated into the supraparticles, which are embedded within the coatings, is progressively released during the coatings’ degradation. The greatest Cu release should occur immediately after implantation to prevent biofilm formation, but the antibacterial effect should be sustained throughout the entire period until the new bone tissue fully replaces the coating. Through testing with MG63 cells, we aimed to assess the biocompatibility of the coatings and with Safe Airborne Antibacterial testing, antimicrobial activity is assessed.

## 2. Materials and Methods

### 2.1. Cu-Doped Supraparticles

#### 2.1.1. Materials for Supraparticle Synthesis and Coating Process

Calcium nitrate tetrahydrate (Ca(NO_3_)_2_ · 4 H_2_O, ≥99%, Sigma-Aldrich, Darmstadt, Germany), ammonium phosphate dibasic ((NH_4_)_2_HPO_4_, ≥99%, Sigma-Aldrich, Darmstadt, Germany), citric acid monohydrate (99.5–102%, Sigma-Aldrich, Darmstadt, Germany), cetyltrimethylammonium chloride solution (CTAC, 25 wt.% in H_2_O, Sigma-Aldrich, Darmstadt, Germany), ethylene glycol (≥99%, Carl Roth, Karlsruhe, Germany), triethanolamine (99%, thermo scientific, Darmstadt, Germany), ethanolamine (≥99%, Carl Roth, Karlsruhe, Germany), ethanol (EtOH, ≥99.5%, Carl Roth, Karlsruhe, Germany), ammonium nitrate (NH_4_NO_3_, ≥98%, Carl Roth, Karlsruhe, Germany), copper nitrate trihydrate (Cu(NO_3_)_2_ · 3 H_2_O, ≥99.5%, Merck, Darmstadt, Germany), nitric acid (HNO_3_, ROTIPURAN^®^ Supra 69%, Carl Roth, Karlsruhe, Germany), β-tricalcium phosphate (Ca_3_(PO_4_)_2_, β-TCP, 97.2%, Chemical Specialist Budenheim, Budenheim, Germany), solution styrene-butadiene rubber (SSBR, 15 wt.% in H_2_O, Targray, Kirkland, QC, Canada), phosphonate based dispersion agent (Zschimmer and Schwarz, Lahnstein, Germany) and hydrocolloid (Zschimmer and Schwarz, Lahnstein, Germany).

#### 2.1.2. Preparation of Cu-Doped CaP Supraparticles (CaPCu and CaPCu HT Particles)

The preparation of CaPCu particles was performed based on the modified sol–gel method described by Höppel et al. [37] In this work, the synthesis of CaP nanoparticles (NPs) was scaled up in a 1 L flask. For this purpose, (NH_4_)_2_HPO_4_ (3.56 g; 26.94 mmol) was added to a mixture of Ca(NO_3_)_2_ H_2_O (7.0 g; 29.64 mmol) and citric acid monohydrate (5.52 g) in deionized (DI) water (460 mL). CTAC (14.8 mL) was then added and stirred at 500 rpm for 10 min. Ethylene glycol (164.45 g) was added and the mixture was cooled to 0 °C for 5 min. After the addition of triethanolamine (164.45 g) and ethanolamine (69.0 g), CaP NPs were precipitated. After stirring for 3 min, the suspension was centrifuged (7800 rpm, 10 min) and redispersed in a mixture of NH_4_NO_3_/EtOH (2.0 wt.%, 300 mL). The suspension was then heated under reflux for 30 min, centrifuged again (7800 rpm, 10 min) and redispersed in EtOH (200 mL). After reheating under reflux for further 30 min, CaP NPs were obtained after centrifugation (7800 rpm, 10 min) and redispersion in DI water (100 mL).

Subsequently, Cu(NO_3_)_2_ · 3 H_2_O (5.0 wt.% relative to CaP) was added to a suspension of CaP NPs and DI water (4.4 wt.%) and the mixture was spray-dried (Buchi B-191; Essen, Germany) at an inlet temperature (T_inlet_) of 130 °C, an inlet hot air flow (aspirator) of 100% and a feed suspension flow (pump) of 15%. CaPCu HT particles were obtained by calcining the CaPCu particles with a heating rate of 2 °C/min at 1000 °C for 15 min (Nabertherm, Lilienthal, Germany).

#### 2.1.3. Preparation of Cu-Doped β-TCP Supraparticles (TCPCu Particles)

For the preparation of spray-drying, the commercial β-TCP microparticles (TCP) were first ground in DI water (1:1) for 1 h at 700 rpm (planetary micro mill PULVERISETTE 7 premium line, FRITSCH, Idar-Oberstein, Germany). Then, a water suspension containing commercially ground TCP (30 wt.%) and Cu(NO_3_)_2_ (5.0 wt.% Cu with respect to β-TCP) was prepared, in which the binder, solution-styrene butadiene rubber (SSBR; 10 wt.% with respect to TCP + Cu(NO_3_)_2_), was added and stirred for 1 h. The mixture was then spray-dried at T_inlet_ = 130 °C, aspirator = 100% and pump = 15%.

### 2.2. Suspensions and Coating Deposition

One of the objectives of this study was to enhance the porosity of bioactive TCP coatings to facilitate accelerated degradation. Thus, preliminary coating experiments were conducted to determine optimal spraying parameters for achieving a porous microstructure. Initially, these experiments utilized only an axially injected β-TCP suspension (preliminary trials). Subsequently, upon the identification of optimal parameters, three distinct Cu-doped supraparticles were introduced to the β-TCP suspension. While the β-TCP serves as the primary constituent for forming the matrix of the resulting coating, the Cu-doped supraparticles, acting as secondary phase, are meant to confer antibacterial properties to the coatings. Additionally, they are expected to increase the microporosity of the coatings. In fact, the supraparticles are not expected to be completely molten during the HVSFS process. As a result, the space between the nanoparticles will be retained, creating micro- or nanoporosity.

#### 2.2.1. Suspensions

For the spraying of the bioconductive coatings, water-based suspensions were used. The β-TCP raw powder was added under continuous stirring into the mixture of DI water with two stabilizing agents: 2 wt.% of a hydrocolloid and 3 wt.% of solid content of a phosphonate-based dispersion agent (see Table S1). For the preliminary trials, the β-TCP powder content was set either to 5 wt.% or to 10 wt.%, to adjust the porosity of the coatings. The β-TCP raw powder had a specification of d_50_ ≤ 10 µm.

For the Cu-doped final coatings, a 5 wt.% β-TCP suspension was prepared as previously described. Subsequently, 0.5 wt.% of the Cu-doped supraparticles were stirred in DI water to prevent agglomeration. This Cu-supraparticles suspension was then added to the β-TCP suspension matrix.

#### 2.2.2. Coating Deposition

A modified Top-Gun-G system (GTV Verschleißschutz, Luckenbach, Germany) was used for this work. The spray gun was mounted on a six-axis robot to perform controlled meander movement with an offset of 3 mm and a spraying distance of 120 mm. Ethen (C_2_H_4_) and oxygen (O_2_) were used for the combustion. All substrates were grit-blasted using F60 corundum with a pressure of 4 bar before coating. Subsequently, they were cleaned with acetone in an ultrasonic bath and then weighed to determine the net weight of the substrate. The relative surface speed was set to 600 mm/s. All coatings were sprayed with a 22-8-135 combustion chamber. Axial pressurized air cooling with two nozzles at the left and right side of the torch axis was used. The samples were also cooled from behind to keep the specimen temperature as low as possible.

The preliminary experiments utilizing solely the β-TCP suspension were conducted on grit-blasted V2A stainless-steel (S) samples (Schmiedekult, rapa GmbH, Emmerich am Rhein, Germany). A total of 10 passes were performed. During these experiments, both the total gas flow (GF)—195 slpm (70 slpm ethen and 125 slpm oxygen) and 230 slpm (80 slpm ethen and 150 slpm oxygene)—and the suspension feed rate (FR)—40 g/min and 80 g/min—were varied. To evaluate the impact of these parameters on the microstructure of the coatings, a total of four samples were produced, each corresponding to different combinations of gas flow and suspension feed rate: 195 slpm and 40 g/min; 195 slpm and 80 g/min; 230 slpm and 40 g/min; 230 slpm and 80 g/min.

For the Cu-doped final coatings, the combination of GF of 195 slpm and FR of 80 g/min was chosen. These coatings were applied to V2A stainless steel specimens (Schmiedekult, rapa GmbH, Emmerich am Rhein, Germany) and Titan (Ti) grade 2 specimens (ARA-T Advance GmbH, Dinslaken, Germany). The stainless-steel specimens were sprayed with 8 passes to achieve thicker layers, thereby facilitating comprehensive analysis of porosity, hardness, phase composition and microstructure. In contrast, the Ti specimens underwent only four passes, aiming for a coating thickness of approximately 30 µm. These Ti specimens were used for biological characterization. Evaluation of their thickness, roughness and Cu content (ICP-MS) was conducted, as these values have an influence on the in vitro results.

All used coating parameter combinations can be found in Table S2.

### 2.3. Particles and Coating Characterization

#### 2.3.1. Scanning Electron Microscopy (SEM) and Energy-Dispersive X-ray Spectroscopy (EDS)

The structure and size of the particles were determined using SEM measurements (Supra 25, Zeiss, Oberkochen, Germany). An acceleration voltage of 1.5 kV and a secondary electron detector was used for this purpose. Prior to the measurements, the samples were prepared on conductive carbon pads and sputtered with platinum for 10 s at 30 mA (MED 010, Balzers Union, Balzers, Liechtenstein).

For the characterization of the coatings, details of the microstructures were observed using a field-emission scanning electron microscope S-800 (Hitachi High-Technologies Corporation, Tokyo, Japan). Cross-section samples were sputtered with carbon before SEM examination. EDS measurements were conducted to determine the Ca to P ratio. Therefore, an ESEM (FEI Quanta 250 FEG, FEI, Hilsboro, OR, USA) equipped with an EDX system (Oxford Instruments INCA x-act, Oxford Instruments, Abingdon, Oxfordshire, Great Britain) was used. The measurements were carried out at 20 kV acceleration voltage over an area of 10 × 10 µm for 5 min (lifetime corrected).

#### 2.3.2. X-ray Diffraction (XRD)

The crystal structure of the particles was determined by XRD (D8 ADVANCE, Bruker, Billerica, MA, USA) using Cu Kα radiation (λ = 1.5406 Å). The diffraction patterns were measured between 7° < 2Θ < 70° with a step size of 0.02°. The phase composition was determined using the software TOPAS 64, version 6.

The phase composition of the coatings was analyzed by X-ray diffraction (XRD: X’Pert PRO, PANAlytical, Almelo, The Netherlands) using Cu–Kα radiation (wavelength: 0.1540598 nm). The diffraction patterns were collected in the 20°–70° 2θ range (step size: 0.02°; scan rate: 5 s/step).

#### 2.3.3. Inductively Coupled Plasma Mass Spectrometry (ICP-MS)

The Cu amounts in the particles and coatings were determined by ICP-MS (iCAP RQ, Thermo Fisher Scientific, Waltham, MA, USA). For this purpose, the samples (3.0 mg) were dissolved in 69% HNO_3_ (1 mL) and diluted with extra-pure water (1:100). The Cu content was measured against a standard solution of 9.8 mg/L. Three measurements were performed per sample and the mean value was calculated.

#### 2.3.4. Vickers Microhardness

The coatings’ Vickers microhardness was measured on polished cross sections using a Fisherscope H100 (Helmut Fischer GmbH Institut für Elektronik und Messtechnik, Sindelfingen, Germany) hardness tester. HV 0.1 scale was used according to DIN EN ISO 14577 standard [38]. The measurement was force-regulated and the applied load of 980.665 mN was performed for 20 s with a load and release time of 5 s. For each sample, 13 imprints on the cross-section of the coating were made and the average hardness and its standard deviation were calculated.

#### 2.3.5. Roughness

The arithmetic mean roughness value R_a_ and the mean roughness depth R_z_ were investigated by tactile measurement with Mahr Perthometer (Mahr GmbH, Göttingen, Germany). The measurement was performed with a length of 17.5 mm and five single measurements according to DIN EN ISO 3274 [39]. For each coating, the values and standard deviation were determined, taking the average values.

#### 2.3.6. Optical Microscopy

Coating microstructures were analyzed through an optical microscope MeF4M (Leica GmbH, Wetzlar, Germany) in bright field. Pictures were taken and analyzed by the software a4i analysis (A4I, London, Great Britain). Coating thicknesses were characterized according to DIN EN ISO 1463:2021-08 by measuring fifteen single coating thickness values and, respectively, calculating average value and standard deviation [40].

### 2.4. In Vitro Characterization

#### 2.4.1. Materials for In Vitro Characterization

TRIS hydrochloride (≥99%, Carl Roth, Karlsruhe, Germany), hydrochloric acid solution (HCl, Honeywell, Morristown, NJ, USA), Dulbecco’s Modified Eagle Medium DMEM/F-12 (DMEM/F12, Gibco, Paisley, Great Britain), Dulbecco’s phosphate buffered saline (DPBS, Gibco, Paisley, Great Britain), fetal bovine serum (FBS, Biochrom, Berlin, Germany), Penicilline/Streptomycin (P/S, Gibco, Paisley, Great Britain), Trypsin-EDTA (Sigma-Aldrich, Darmstadt, Germany), Live/Dead Cell Staining Kit II (PromoCell, Heidelberg, Germany), Cell Proliferation Reagent WST-1 (Roche, Basel, Switzerland), Cytotoxicity Detection Kit (LDH) (Roche, Basel, Switzerland), Tryptone Soya Broth (TSB, Oxoid, Wesel, Germany), 0.9% Sodium chloride (NaCl) solution (B. Braun, Melsungen, Germany), Columbia agar plates (Oxoid, Wesel, Germany), Sodium chloride (NaCl, ≥99.5%, Carl Roth, Karlsruhe, Germany), Sodium Hydrogen Carbonate (NaHCO_3_, ≥99.5%, Carl Roth, Karlsruhe, Germany), Potassium Chloride (KCL, ≥99, Sigma-Aldrich, Darmstadt, Germany), Di-Sodium-Hydrogen Phosphate Dihydrate (Na_2_HPO_4_2H_2_O, E., ≥99.5%, Merck, Darmstadt, Germany), Magnesium Chloride (MgCl_2_, ≥98%, Sigma-Aldrich, Darmstadt, Germany), Calcium Chloride Dihydrate (CaCl_2_2H_2_O, 98%, Sigma-Aldrich, Darmstadt, Germany), Sodium Sulfate (Na_2_SO_4_, ≥98%, Carl Roth, Karlsruhe, Germany) and Trizma^®^ Base ((CH_2_OH)_3_CNH_2_, ≥99.9%, Carl Roth, Karlsruhe, Germany) MG-63 cells (ATCC, CRL 1427, Manassas, VA, USA), *Staphylococcus aureus* (*S. aureus*, ATCC29593, Manassas, VA, USA) and *Escherichia coli* (*E. coli*, ATCC29522, Manassas, VA, USA).

#### 2.4.2. Preparation of the Samples

Prior to the in vitro experiments, the samples were disinfected in 70% and 100% ethanol. After that, they were autoclaved using the Systec D-Series Horizontal Benchtop autoclave (Thermo Fisher Scientific, Waltham, MA, USA).

#### 2.4.3. Eluent Experiments

Following ISO standard 10993-15:2019-11, three samples of each coating were placed into Thermo Scientific 15.0 mL tubes [41]. The samples were incubated in Tris buffer (6 mL), which was adjusted to pH 7.4 using 1 M HCl. The incubation was carried out for 5 days and the samples were kept at a temperature of 37 °C. After 30 min, 24, 48, 72, 96 and 120 h, the samples were transferred to new 15 mL tubes and new Tris buffer (6 mL) was added. The collected solutions per day and the samples were separately analyzed using an atomic absorption spectrometer (Perkin Elmer AAS 4110ZL Zeeman, Perkins Elmer, Waltham, MA, USA) at the Institute of Geosciences, University of Freiburg, Germany.

#### 2.4.4. Biocompatibility Testing In Vitro

Biocompatibility testing was conducted using MG-63 cells. All experiments were repeated at least three times. The cells were maintained in a medium composed of DMEM/F-12, 10% FBS and 1% P/S. The cells were passaged twice a week by Trypsin/Ethylene diamine tetraacetic acid treatment and kept at 37 °C, 5% CO_2_ and 100% humidity. The medium was changed every other day.

All tests were performed using 50,000 cells/75 µL per sample. Additionally, cells seeded onto a Thermanox^®^ cover slip were used as a control (C-). After cell seeding was performed, the well plates were incubated for 2 h at 37 °C and at a CO_2_ saturation of 5%.

#### 2.4.5. Live/Dead Assay

After the 2 h incubation period, specific cell medium (1 mL), as previously described, was added to each well. The well plates were then incubated for 1, 3 and 7 days. For staining, a solution was prepared as suggested in the work of Burtscher et al. [7]. To remove serum esterase activity, all samples were washed three times using DPBS. Then, the staining solution (600 µL) was added and incubated for 10 min. Afterwards, the samples were transferred into a well plate containing DPBS. The evaluation was conducted using an Olympus fluorescence microscope (BX51, Olympus, Osaka, Japan) at four different positions on the samples, including one overview and three detailed images at 5× and 10× magnification. All steps were carried out in the dark to prevent photobleaching.

#### 2.4.6. Cell Proliferation (WST-I)

After the 2 h incubation period, DMEM-F12 (1 mL), along with 1% P/S and 1% FBS, was added to all the wells. It has to be noted that a higher concentration of FBS can lead to background absorption, which is why only 1% FBS was used in this experiment. The cell proliferation assay was carried out in accordance with the manufacturer’s instructions, as described in our previous work [42]. Then, 10% WST solution was added to all the samples at designated measurement time points (1, 3 and 7 days) and allowed to incubate for 2 h. Following that, the absorbance was measured at 450 nm using a spectrometer (SpectroStar nano, BMG Labtech, Ortenberg, Germany). To avoid photoactivation, the experiment was carried out in the dark.

#### 2.4.7. Cytotoxicity (LDH Assay)

The cytotoxicity measurements were carried out after 24, 48 and 72 h. In addition to negative controls, positive controls (C+, cells on a Thermanox cover slip + Triton X, 100% toxicity) were used for the measurements at all time points. After the 2 h incubation period, the wells were filled up (1 mL) using DMEM-F12 with 1% P/S and 1% FBS added. At each measurement time stamp, medium (100 µL) from each well was transferred to 3 new wells of a 96-well plate. To prepare the cytotoxicity detection kit solution, the catalyst solution and staining solution were mixed in a ratio of 1:45. The solution (100 µL) was pipetted into each well. After 30 min incubation, absorbance at 490 nm was measured using a spectrometer (SpectroStar nano, BMG Labtech, Ortenberg, Germany).

#### 2.4.8. Antimicrobial Testing

To evaluate the antimicrobial properties, Safe Airborne Antibacterial Assays (SAAA) were performed using *S. aureus* and *E. coli*. The approach followed the methodology of Al-Ahmad et al., with a detailed description of the experimental setup available elsewhere [43]. Here, the assay was performed as described in the work of Burtscher et al. [7].

#### 2.4.9. Hydroxyapatite Formation in Simulated Body Fluid (SBF)

The Simulated Body Fluid (SBF) was prepared following the procedure outlined by Jalota et al. [44]. The solution’s pH was adjusted to 7.4 using a 1 M HCl solution. A set of 3 samples was then placed in a 24-well plate, each covered with SBF solution (2 mL). Following a 14-day incubation period at 37 °C, the samples were rinsed with DI water and allowed to air-dry. Evaluation of HAp formation was conducted using electron microscopy at the Freiburg Materials Research Center in Freiburg, Germany. Additionally, XRD measurements were carried out to confirm the presence of HAp.

### 2.5. Statistics

The data were expressed as mean ± standard deviation and subjected to one-way analysis of variance (ANOVA). Mean values were compared using Fisher LSD. A significance threshold of *p* < 0.05 was applied. Calculations were conducted using OriginPro 2023 SR1 (OriginLabs, Northampton, MA, USA).

## 3. Results and Discussion

### 3.1. Cu-Doped Self-Synthesized CaP and Commercial β-TCP Supraparticles

The preparation of the Cu-doped CaP supraparticles was performed as described in a previous work [37]. Figure S1A shows the prepared Cu-doped supraparticles (CaPCu particles) which have a spherical structure with a size of 1–12 µm [45]. In this context, low-crystalline HAp was formed (Figure S1C). A part of the supraparticles was then calcined (CaPCu HT particles), resulting in crystalline phases of HAp (≈70%) and β-TCP (≈30%) as well as CuO (Figure S1C). The use of biphasic CaP offers advantages over monophasic CaP, such as increased osteoinduction and biodegradability [46]. In addition, the Cu amount increased from 5.0 to 6.9 wt.% due to the thermal decomposition of the organic substances during calcination, as shown in the study by Höppel et al. [37]. However, calcination led to the aggregation of the supraparticles as well as of the primary NPs (Figure S1B), resulting in a significant decrease in porosity [37]. Therefore, both CaPCu and CaPCu HT particles were used in the further experiments for comparison.

In this study, the synthesis of CaP NPs has been scaled-up from the previous work by Höppel et al. in a 1 L flask, but is still accompanied by a low yield (≈3.0 g) [37]. Due to losses in the glass apparatus, spray-drying also results in a low yield (≈60%). In addition, the subsequent calcination to obtain crystalline phases causes mass losses of organic components (yield: ≈73%) [47]. Therefore, the use of commercial particles for later applications and the possibility of mass production is preferable. On the one hand, this reduces effort and costs. On the other hand, crystalline structures are obtained directly by using crystalline β-TCP particles. This avoids the calcination step with its mass losses and the aggregation of the supraparticles.

**Figure 1:**
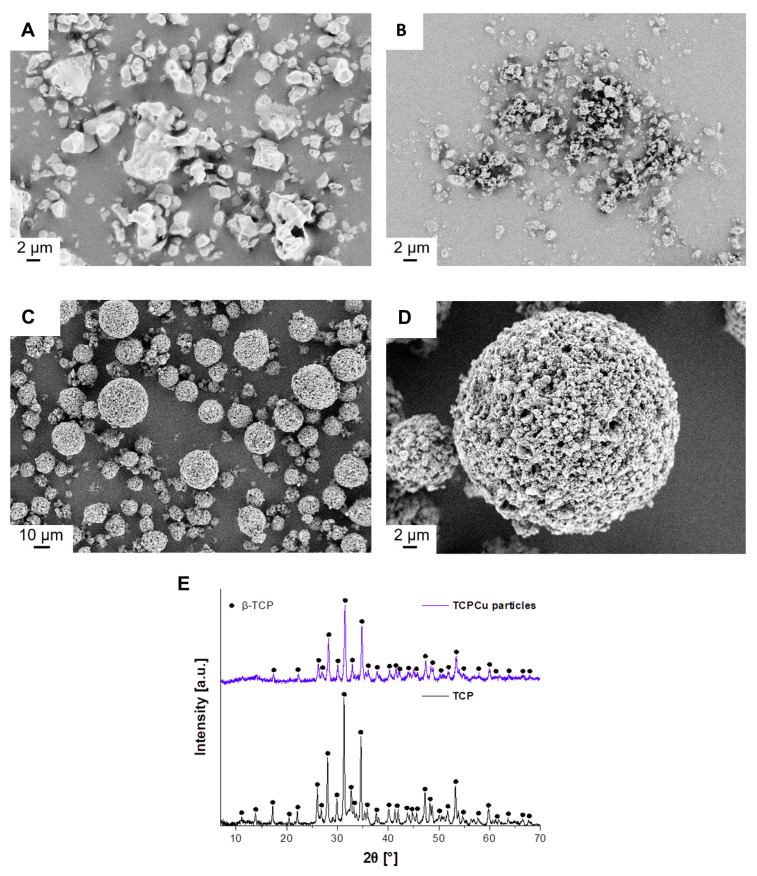
(**A**) SEM image of commercial β-TCP microparticles (TCP) with a size of 0.7–18 µm. (**B**) SEM image of commercially grinded TCP with a size of 0.1–8 µm. (**C**) SEM image of the spherical supraparticles prepared on the basis of commercial TCPCu particles with a size of 1–34 µm and (**D**) section of a TCPCu particle. (**E**) XRD pattern of TCP (bottom) and TCPCu particles (top) indicating no differences before and after spray-drying.

For this reason, commercial β-TCP microparticles (TCP) with a size of 0.7–18 µm were tested in addition to the self-synthesized CaP NPs (Figure 1A). Prior to spray-drying, the particles were ground for 1 h, resulting in a size of 0.1–8 µm (Figure 1B). The use of smaller primary particles allows the sufficient attachment of the particles in a droplet, which should favor the complete formation of supraparticles and a higher stability of the supraparticles. In addition, the binder SSBR was used to ensure strong binding adhesion between the primary particles. The spray-drying of grinded TCP (40 wt.%) together with Cu ions (5.0 wt% based on β-TCP) and SSBR (10 wt.% based on β-TCP + Cu(NO_3_)_2_) was then performed, resulting in spherical supraparticles (TCPCu particles) with a size of 1–34 µm and a yield of 53% (Figure 1C,D). After spray-drying, no loss of Cu ions (5.0 wt.%) was observed, as measured by ICP-MS. The crystal structure also remained unchanged and β-TCP was still completely obtained after spray-drying with Cu ions. In addition, no reflections of the Cu(NO_3_)_2_ were found, as already observed with the CaPCu particles (Figure 1E). The formation of larger supraparticles (1–34 µm) compared to the CaPCu particles (1–10 µm) was a consequence of the higher β-TCP concentration used (40 wt.%) [45]. Thus, not only particle types with different crystal structures (low crystalline HAp, HAp/β-TCP, β-TCP), but also different supraparticle sizes (1–12 µm and 1–34 µm) can be compared with each other for further experiments.

### 3.2. Suspensions Used for Coating Experiments and Properties of the Deposited Coatings

**Figure 2:**
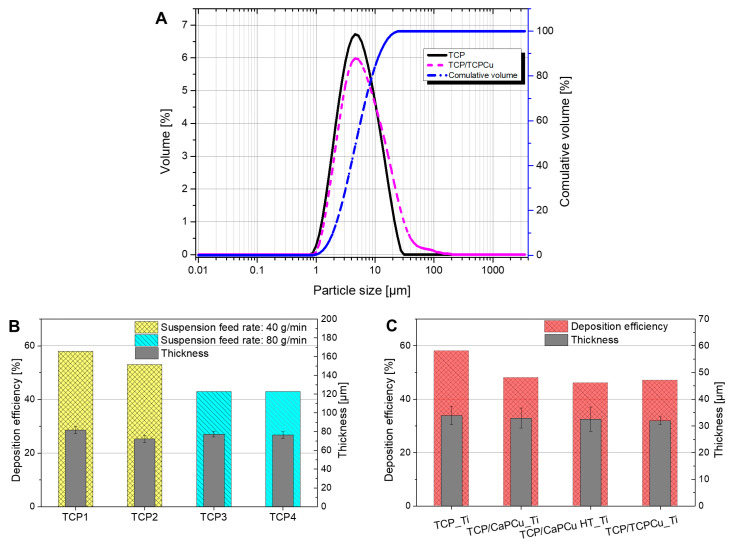
(**A**) Particle size distribution of the TCP and TCP/TCPCu suspensions. The cumulative volume is the same for both suspensions. (**B**) Deposition efficiency and thickness of the pre-trial coatings. TCP1 and TCP2 were sprayed with a feeding rate of 40 g/min, whereas TCP3 and TCP4 with an 80 g/min feeding rate. (**C**) Deposition efficiency and thickness of the TCP reference sample and TCP with different Cu-doped supraparticles samples on Ti substrate (80 g/min feeding rate).

The suspension used shows a monomodal particle size distribution. Particles are well distributed, there is no agglomeration and the d_50_ is 5.4 µm, whereas the d_90_ is 13.7 µm (see Figure 2A). These values are in line with the raw powder specifications mentioned in Section 2.2.1. Figure 2A also shows an example for the suspension of TCP and Cu-doped supraparticles (TCP/TCPCu). The particles, as described in the previous chapter, have a size of 1–34 µm. The particle size distribution grows thus as expected to a d_50_ of around 6 µm and d_90_ of 20 µm. A minor shoulder can be seen on the right side of the curve. This should be because of the supraparticles, or it could be a sign of slight agglomeration. All suspensions could be sprayed without clumping the feeding system.

The deposition efficiency (DE) of the preliminary trials conducted to find optimal parameters for a porous microstructure can be seen in Figure 2B. The highest DE is found for the lower gas flow parameter at a smaller axial feed rate of 40 g/min (TCP1). The higher suspension feed rate reduces the DE of the coating process (TCP3 and TCP4). Their thickness is, on the contrary, not affected by the different feeding rates and all the samples show similar thicknesses of around 80 µm. This is probably because the thickness results must be considered also in relation to the porosity, since a higher porosity always results in apparently thicker coatings [48].

The supraparticles have a small influence on the DE of the coatings sprayed on Ti substrates (Figure 2C). The reference sample of pure TCP has a higher DE of 58% compared to all the coatings with a second phase. It is also higher than the sample, which was sprayed for the preliminary trials and 10 passes, which had a DE of 53%. The coatings with the supraparticles achieve a similar DE (TCP/CaPCu 48%, TCP/CaPCu HT 46% and TCP/TCPCu 47%). The coating thicknesses show no significant difference and range between 31.95 ± 1.5 µm (TCP/TCPCu) and 33.9 ± 3.43 µm (TCP).

**Figure 3:**
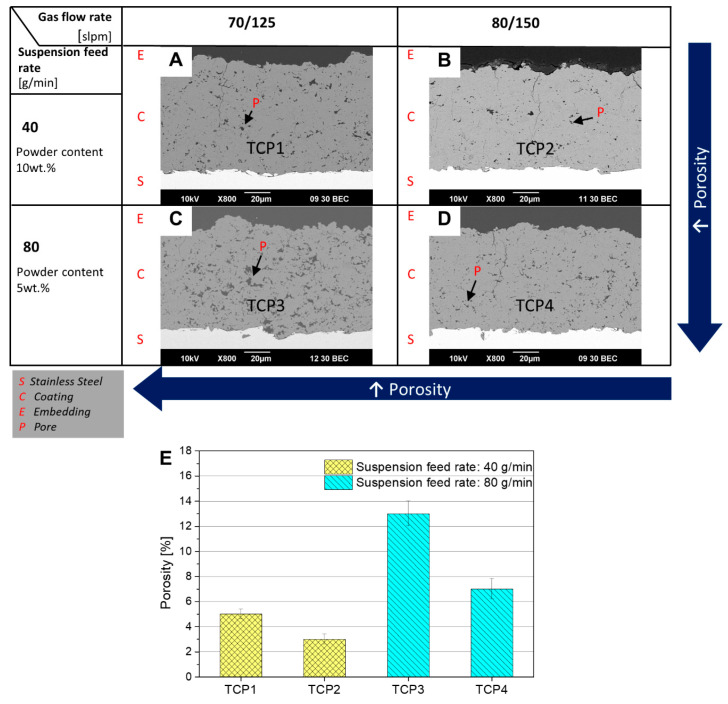
SEM pictures of pre-trial coatings (**A**) TCP1, (**B**) TCP2, (**C**) TCP3 and (**D**) TCP4. The porosity increases with falling gas flow parameters and increasing suspension feed rate. The powder content was reduced by half to keep the thicknesses comparable. (**E**) Porosities of preliminary trials’ sprayed samples.

The SEM images of the parameter study are shown in Figure 3. The porosity grows with decreasing gas flow parameters and an increasing suspension feed rate.

TCP2 shows the lowest porosity (3%) followed by TCP1 (5%). Sample TCP3, which was sprayed with the lowest gas flow and highest suspension feed rate parameters, achieves a porosity of around 13%. TCP4 shows smaller pores and a total porosity of 7%. The porosities can be seen in Figure 3E. Since a porosity greater than 9% was set as the target, the parameters of sample TCP3 were selected for further investigations with integrated Cu-doped supraparticles. For this final trial, a total of four samples were produced: one reference sample (TCP) and three samples with different Cu-doped supraparticles (TCP/CaPCu, TCP/CaPCu HT and TCP/TCPCu). The differences between the particles are described in the Section 3.1.

**Figure 4:**
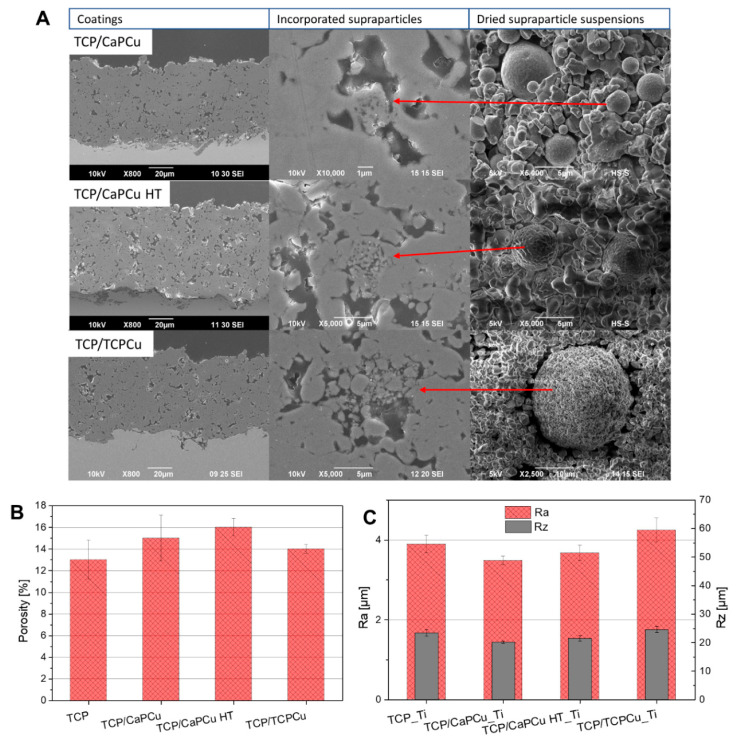
(**A**) SEM pictures of the sprayed coatings on S samples with integrated supraparticles. The pictures presented on the right exhibit the dried supraparticle suspensions, serving to elucidate the morphological disparities between the particles pre- and post-spraying of the TCP/supraparticles coatings sprayed on S samples. (**B**) The porosities of the samples, where the supraparticles seem to increase the microporosity of the layers. (**C**) Roughness values Ra and Rz of the coating sprayed on Ti substrates.

SEM images of the coatings with incorporated Cu supraparticles are shown in Figure 4A. Here, the supraparticles seem to create voids between the matrix splats. This way, they seem to improve the microporosity of the coatings, which increases up to 16% for the TCP/CaPCu HT coating. The reference TCP coating shows the less porous microstructure (13%). All porosity results of the coatings are shown in Figure 4B.

The roughness R_a_ of the sprayed coatings ranges between 3.48 µm and 4.24 µm. R_z_ values are between 20.19 µm and 24.65 µm. The results are shown in Figure 4C. In comparison, plasma-sprayed coatings have roughness values in the range of 1.06 ± 0.64 µm [49].

The supraparticles do not change the roughness of the coatings. In general, it is difficult to say which roughness is good for osteointegration and bone healing [50]. For dental implants, a roughness of R_a_ > 2 µm is categorized as maximal [50]. Higher roughness should help the cells to adhere and anchor to the coating, so that biomechanical stability is achieved [51]. Wennerberg and Albrektsson [52] stated that a higher R_a_ than 1–1.5 µm would lead to the deterioration of implant fixation. Too high a surface roughness may increase the risk for peri-implantitis as well. However, the study of Schwarz et al. [51] as well as other studies disprove the assessment of Wennerberg and Albrektsson. They note that bone loss is caused by many factors and the clinical impact of surface roughness alone on bone loss and peri-implantitis is of minimal clinical importance [51,53].

**Figure 5:**
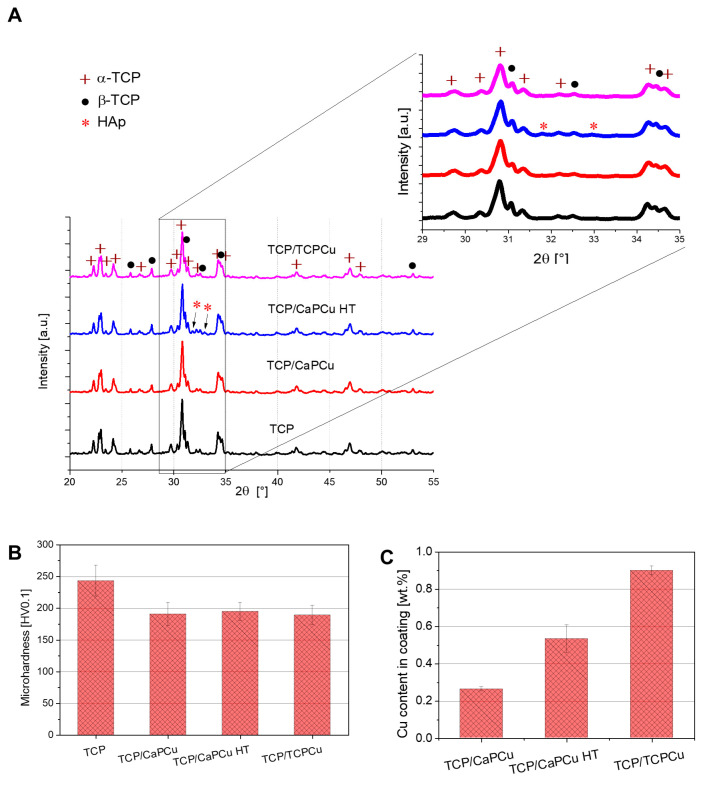
(**A**) XRD spectra of TCP coatings. TCP is present in α- and β-form. Through the implementation of the supraparticles, only a change in the TCP/CaPCu HT coating can be observed, where also a minor peak of HAp could be detected. (**B**) The microhardness of the S samples. The reference specimen TCP shows the highest microhardness. The porosity caused by the introduction of the supraparticles seems to decrease the microhardness of the coatings. (**C**) ICP-MS measurement of the TCP coatings with Cu-doped supraparticles.

The microhardness results are shown in Figure 5B. The TCP reference sample without supraparticles shows the highest hardness of 243.2 HV0.1, which is in line with TCP coatings in the literature [54]. The introduction of supraparticles into the coating layers causes a reduction in the hardness. This trend can be explained by the increase in porosity and is confirmed by the literature [55].

An XRD analysis of the coatings shows the phase composition and crystallinity of the coatings’ surface (see Figure 5A). As can be seen, the raw β-TCP powder underwent no significant changes in the phase composition. The gas parameters are thus suitable for this material. In the coatings, mainly TCP, in α- and β-form was detected. α-TCP is the high-temperature phase while β-TCP is the low-temperature phase. An XRD analysis of the powder showed the presence of calcium pyrophosphate (CPP). The phase composition is 90.5 ± 0.45% β-TCP (reference pattern PDF# 04-010-4348) and 9.5 ± 0.45% CPP (reference pattern PDF# 04-009-3876). In the coatings, mainly α-TCP (reference pattern PDF# 04-010-4348) can be seen in the XRD analysis, which represents 76.76 ± 0.23% of the phase composition for reference sample TCP7, while 17.45 ± 0.19% is βTCP and 5.79 ± 0.18% is CPP. The CPP peak is at 28.9°. For the sample with CaPCu supraparticles, α-TCP represents 75.74 ± 0.31%, β-TCP 14.98 ± 0.21% and CPP 9.28 ± 0.28%; for the TCPCu particles, the phase composition is 71.96 ± 0.28% α-TCP, 16.88 ± 0.19% β-TCP and 11.16 ± 0.26% CPP. The α-TCP phase is probably formed because of the high temperature reached by the particles when injected into the HVSFS flame. The CPP phase was already present in the powder. These phases are faster to degrade than β-TCP but are not cytotoxic and should not impair the coating’s properties [20]. Only in the TCP/CaPCu HT coating, a HAp phase was also found. Here we have a phase composition of 71.24 ± 0.29% α-TCP, 18.28 ± 0.20% β-TCP, 7.14 ± 0.30% CPP and 3.33 ± 0.13% HAp (reference pattern PDF# 01-076-0694). This was to be expected since the CaPCu HT supraparticles also showed HAp peaks (see Figure S1C). We additionally carried on EDS on the surface of our coatings and from the results we could measure the Ca/P ratio. The reference sample TCP7 has an atomic ratio of 1.45, while the Ca/P ratio of the coatings with supraparticles varies from 1.44 to 1.46 (TCP/CaPCu 1.45; TCP/CaPCu 1.46; TCP/TCPCu 1.44). A ratio of less than 1.5 in TCP indicates that it is non-stoichiometric or calcium-deficient TCP. This is due to the presence of CPP in the phase [56].

ICP-MS shows the presence of Cu in the coated layers. As can be seen in Figure 5C, the amount varies for the different supraparticles used. The TCP/TCPCu (0.9 ± 0.02 wt.% Cu) coating has the highest amount of Cu, followed by TCP/CaPCu HT (0.53 ± 0.08 wt.% Cu) and TCP/CaPCu (0.26 ± 0.01 wt.% Cu). Since the process and suspension parameters were kept the same for all the samples, these results might be caused by the used supraparticles. As described above, the TCPCu supraparticles are larger than the self-synthesized CaPCu supraparticles. The higher surface volume could lead to an increase in the total Cu amount in the layers. In addition, because of the higher surface volume, the supraparticles survive the high temperature in the gas flame better and are less molten. The calcinated CaPCu supraparticles have a higher amount of Cu than the CaPCu supraparticles (6.9 wt.% instead of 5.0 wt.%), as described in the previous subsection. This likely explains why the Cu content is higher in the final coating containing CaPCu HT particles compared to the coating with CaPCu supraparticles.

### 3.3. In Vitro Tests

#### 3.3.1. Eluent Experiment

The Cu release of the samples containing Cu is between 2.22 mg/L and 5.85 mg/L after 120 h. The exact values can be found in Table 1. For all samples, the largest Cu release takes place within the first 24 h, with TCP/CaPCu presenting with a notable burst release during the first 24 h of testing. After that, the release becomes significantly lower for all samples and approaches a plateau.

The Cu release correlates with the amount of Cu in the coating measured via ICP-MS. The total release for TCP/CaPCu is the lowest with 2.22 mg/L after 120 h, while ICP-MS shows the lowest Cu value for this coating. Figure S2 further demonstrates a higher amount of Cu for TCP/CaPCu HT than for TCP/TCPCu. Similar results can be found in the eluent experiment. TCP/CaPCu HT presents with the highest Cu release with 5.85 mg/L, while the release for TCP/TCPCu is lower with 3.93 mg/L. The Cu content measured via ICP-MS was discussed previously. Besides the Cu content in the coating, it can be assumed that the different compositions of the supraparticles may have an influence on the solubility of the coating and, therefore, on the Cu release, too. An XRD analysis of the particles showed the presence of low-crystalline HAp for uncalcined CaP supraparticles and a mixture of HAp and ß-TCP for calcined CaP supraparticles. Furthermore, other coating properties such as porosity and hardness have to be taken into account when looking at the dissolution rate of the coating [57,58]. Generally, higher porosities lead to the better dissolution of coatings since the mechanical integrity of the coating is lower and liquid can, therefore, permeate the coating more easily, leading to a better solubility [58]. Hardness also plays a crucial role when it comes to the degradation of a coating. For harder coatings, it is more difficult to break the intermolecular interactions which results in lower solubility [59].

#### 3.3.2. Live/Dead Assay

**Figure 6:**
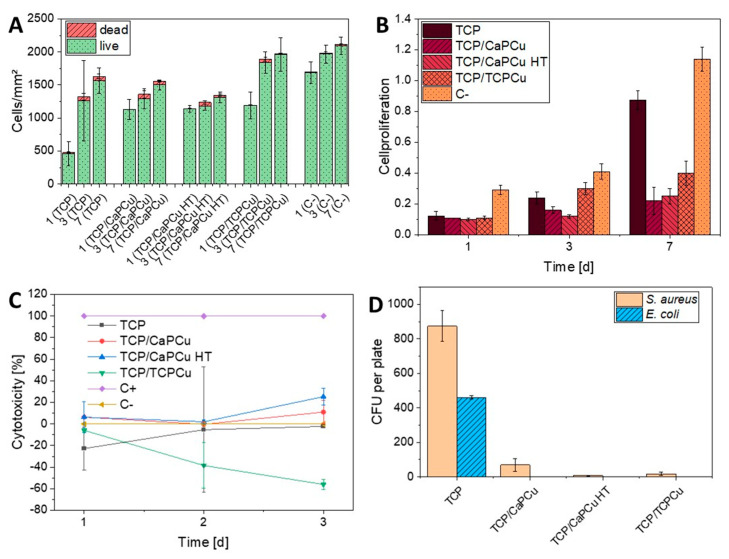
(**A**) Live and dead cells after 1, 3 and 7 days with cells on Thermanox^®^ membrane as C-. (**B**) Cell proliferation on day 1, 3 and 7. Medium with cells on a Thermanox^®^ membrane serves as C-. (**C**) Cytotoxicity on day 1, 2 and 3. Triton X on cells serves as C+, medium with cells serves as C-. (**D**) Number of Colony-forming units (CFU) per plate after a 30 min incubation period.

All samples tested show similar live/dead cell counts and similar proliferation kinetics over 7 days, which can be seen in Figure 6A. Under the microscope, live cells present themselves green while dead cells appear red.

All TCP samples show an increasing number of alive cells within 7 days, while the number of dead cells stays below 100 cells/mm^2^. Yet, the numbers of dead cells are slightly higher for the TCP-coated samples than for C-.

On day 1, all samples show a lower amount of living cells on the coatings compared to C-. After 7 days, TCP/TCPCu presents with an alive cell count similar to C-, while the other coatings have a slightly lower number of alive cells. TCP/CaPCu HT shows the lowest quantity of living cells, TCP and TCP/CaPCu present with a slightly higher number of alive cells.

#### 3.3.3. Cell Proliferation (WST-I)

Figure 6B shows the cell proliferation rate over 7 days. In comparison to C-, all TCP samples show a significantly slower cell proliferation rate. The sample containing no Cu shows increasing proliferation between day 3 and 7 with further proliferation tendencies. Both TCP/CaPCu and TCP/CaPCu HT show very little proliferation during the time period tested. TCP/TCPCu shows an increase in cell proliferation until day 3, after that the cell proliferation subsides until day 7.

#### 3.3.4. Cytotoxicity (LDH)

As shown in Figure 6C, both TCP and TCP/TCPCu show cytotoxicity values below C-. TCP/CaPCu presents with a cytotoxicity value of 11.05% on day 3 and TCP/CaPCu HT shows a value of 25.34%.

Several studies have shown excellent biocompatibility for ß-TCP-coatings in both in vitro and in vivo studies [60,61]. Also, CaP particles themselves seem to have a good biocompatibility due to their similarity to human bone [62]. Similar results can be found in this study: the biocompatibility testing shows good results for all coatings tested. Live/Dead staining shows a large number of viable cells and a low number of dead cells, while the cytotoxicity testing only shows positive results for TCP/CaPCu and TCP/CaPCu HT. The WST assay shows an increase in cell proliferation over seven days especially for TCP/TCPCu and the Cu-free sample TCP. The biocompatibility testing shows that the amount of Cu released plays a significant role in the biocompatibility. The results show decreasing amounts of alive cells with increasing Cu release. For instance, the number of viable cells is lowest for TCP/CaPCu HT while the Cu release is the largest for this sample. The correlation between the Cu release and biocompatibility has been investigated by Unabia et al. They found a cytotoxic threshold at 3 mol% for Cu-doped HAp coatings. Increasing the Cu concentration led to a drastic reduction in alive cells [63].

It has to be noted that there are slight discrepancies within the results of the biocompatibility testing. While Live/Dead staining barely shows dead cells, LDH testing states cytotoxicity of 11% for TCP/CaPCu and 25% for TCP/CaPCu HT on day 3. This could be explained by the principle of LDH testing. Since it does not allow additional feeding, cells might die due to a lack of nutrients resulting in a positive cytotoxicity value. Thus, the role of this result can be rated as minor. Furthermore, DIN EN ISO 10993 only considers materials with values over 30% in LDH testing to be cytotoxic [41,64]. Another conspicuous feature can be seen in the WST results. While Live/Dead staining shows cell numbers on the coatings closer to the negative control, WST states a much higher cell proliferation for C- than for the coated samples. A possible reason for that finding might be Cu interference with WST-1. Semisch et al. stated an interaction of Cu with the reduction of WST-8 [65]. Another reason that could explain the difference between Live/Dead and WST results might be the underestimation of living cells in Live/Dead staining on C- as the multi-layer formation of the cells after 7 days of incubation makes it difficult to count the cells.

#### 3.3.5. Antibacterial Safe Airborne Assay

All samples containing Cu show a good antibacterial effect. The antibacterial efficacy of Cu against both Gram-positive and Gram-negative bacteria has been proven in several works already [64,66]. Also, the implementation of metal ions in different particle systems to obtain an antibacterial effect of the coatings has been described [67]. The main mechanisms behind bacteria killing by Cu are the membrane damage of the bacteria as well as the formation of reactive oxygen species, which among others, leads to DNA damage [68,69].

As Figure 6D shows, the addition of 0.5 wt.% Cu-doped supraparticles significantly reduces the amount of colony-forming units (CFU) for both bacteria strains compared to the sample containing no Cu. The addition of Cu leads to the eradication of *E. coli* on all coatings. For *S. aureus*, TCP/CaPCu HT shows the best antibacterial effect, while there still is slight bacteria growth on TCP/TCPCu and growth up to 70 CFU on TCP/CaPCu. It can be assumed, that the antibacterial efficacy is strongly linked to the amount of Cu released [70]. TCP/CaPCu released the lowest amount of Cu over the course of 120 h while showing the lowest antibacterial effect compared to the other coatings containing Cu. On the other hand, TCP/CaPCu HT shows the highest Cu release while exhibiting the highest antibacterial effect. All in all, Cu coating appears to reduce the initial microbial adhesion, which is considered the first stage of biofilm formation [71]. Thus, Cu coatings can prevent biomaterial-associated and harmful biofilm infections.

#### 3.3.6. Hydroxyapatite (HAp) Formation in Simulated Body Fluid (SBF)

After 14 days of immersion in SBF solution, structural changes could be detected in all samples (see Figure S3). Electron-microscope imaging showed the formation of crystalline structures, suggesting a new formation of HAp. XRD measurements confirmed the formation of HAp. The density of the newly formed HAp crystals varies for the different coatings. Microscopically, TCP/CaPCu HT shows the largest amount of newly formed crystals throughout the whole surface of the coating, whereas in the other samples, new HAp crystals can only be found in some places. HAp formation depends on the dissolution of the coating. It has been described that high-crystalline coatings are stable in SBF, while a lower crystallinity leads to a faster dissolution of the coating. This resolves in supersaturation with the Ca and P of the surrounding solution and re-precipitation of new HAp crystals [72]. Considering this finding, the different density of newly formed HAp crystals on our coating can be explained by the different dissolution behaviors of our coatings which were discussed earlier.

While SBF experiments are an established way to investigate the in vitro bioactivity/bioconductivity of biomaterials, it has to be noted that there are limitations [73]. SBF simulates the ion concentrations of human blood plasma but neglects the existence of proteins and the role of the musculoskeletal system in bone formation on the coated implants. Secondly, our SBF experimental setup constitutes a closed system while the dissolution and re-precipitation process in vivo takes place in an dynamic and open system, which needs to be taken into account when evaluating our experiment [74]. Therefore, the in vivo performance of the coatings can only be predicted to a limited extent [75].

## 4. Conclusions

This study demonstrates the feasibility of using HVSFS to fabricate thin, biocompatible and porous coatings. Initially, we enhanced the porosity by adjusting the gas and suspension parameters, achieving an up to 13% increase. Subsequently, by incorporating 0.5 wt.% of CaP or TCP supraparticles into the layers, we reached up to 16% porosity. Additionally, we established that doping the CaP supraparticles with 5 wt.% Cu ions confers antibacterial activity while preserving the coatings’ biocompatibility in all samples. No relevant cytotoxicity could be detected, while cell growth was not significantly reduced in our samples. Overall, TCP/TCPCu showed the best results in both biocompatibility and antibacterial testing. While the growth of both *S. aureus* and *E. coli* is nearly fully suppressed, cell numbers and proliferation kinetics are the best compared to all samples tested. To validate if porosity has a real impact on the degradation behavior of the coatings, in vivo testing with the TCP/TCPCu coatings will be done in the future. Ti grade 2 rod samples will be coated and implanted in the femur of rabbits.

Based on these results, future investigations will explore the use of axial/radial injections for such bioconductive coatings. Through radial injections, the supraparticles experience less thermal stress, since the dwell time in the hot flame is reduced. With this approach, supraparticles loaded with drugs, such as antibiotics, could be sprayed. The drug in the core of the supraparticles should be protected by their shell and the supraparticles could be used as a drug delivery system.

## Figures and Tables

**Table 1:** Cumulative Cu release within 120 h [mg/L].

	0.5 h	24 h	48 h	72 h	96 h	120 h
TCP/CaPCu	0.7	1.34 ± 0.05	1.5 ± 0.01	1.64 ± 0.03	1.9 ± 0.04	2.22 ± 0.01
TCP/CaPCu HT	2.5	4.21 ± 1.73	4.62 ± 0.03	5.0 ± 0.01	5.65 ± 0.03	5.85 ± 0.01
TCP/TCPCu	0.93	1.45 ± 0.18	2.31 ± 0.08	3.33 ± 0.12	3.8 ± 0.09	3.93 ± 0.01

## Data Availability

All data supporting the findings of this study are available from the corresponding author upon reasonable request.

## Supplementary Materials

The following supporting information can be downloaded at: https://www.mdpi.com/article/10.3390/jfb15100281/s1, Figure S1: Characterization of CaPCu supraparticles before and after calcination. SEM image of CaPCu particles (A) and CaPCu HT particles (B) with a size of 1–12 µm. (C) XRD pattern of CaPCu particles (bottom) and CaPCu HT particles (top); Figure S2: Cumulative Cu release within 120 h [mg/L]; Figure S3: SEM micrographs of coatings before (A) and after 14-day immersion in SBF, TCP/CaPCu (B), TCP/CaPCu HT (C), TCP/TCPCu (D). Table S1: Summary of formulation of aqueous (DI water) suspension with additives used; Table S2: Overview of the coating parameters.
