# Supplementary material for: Suspension-Sprayed Calcium Phosphate Coatings with Antibacterial Properties
Source: J Funct Biomater. 2024 Sep 25;15(10):281. doi: 10.3390/jfb15100281 (PMC11509040; doi:10.3390/jfb15100281)
Supplement: Supplementary file 1 [file jfb-15-00281-s001.zip › jfb-3190268-supplementary.pdf]

Supplementary data

Table S1. Summary of formulation of aqueous (DI water) suspension with additives used.

Material	Manufacturer	Used additives Type:	Content [wt.%]	Solid content [wt%]
β-TCP (+ supra-particles)	Budenheim	-Phosphonate based dispersant	3 wt.% of solid content	5 or 10
		-Hydrocolloid	2 wt.% of total suspension	

Table S2. Overview of the coating parameters.

Coating denotation	Suspension feed rate [g/min]	Total gas flow [slpm]	C_2H_4 [slpm]	O_2 [slpm]	Passes [x]
TCP1	40	195	70	125	10
TCP2		230	80	150	10
TCP3	80	195	70	125	10
TCP4		230	80	150	10
TCP	80	195	70	125	4(Ti)/8(SS)
TCP/TCPCu					
TCP/CaPCu					
TCP/CaPCu HT					
Ti = Titan, SS = Stainless Steel					

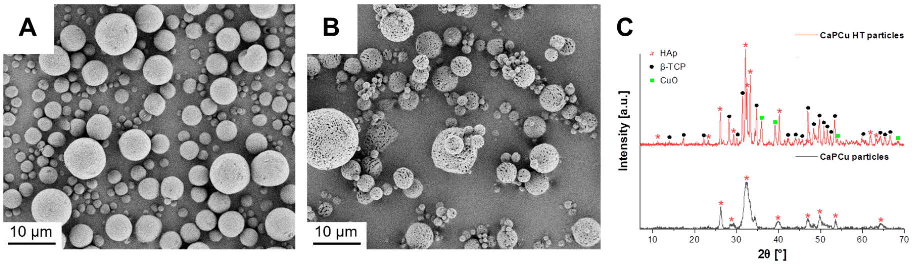

Figure S1. Characterization of CaPCu supraparticles before and after calcination. SEM image of CaPCu particles (A) and CaPCu HT particles (B) with a size of 1 – 12 µm. (C) XRD pattern of CaPCu particles (bottom) and CaPCu HT particles (top).

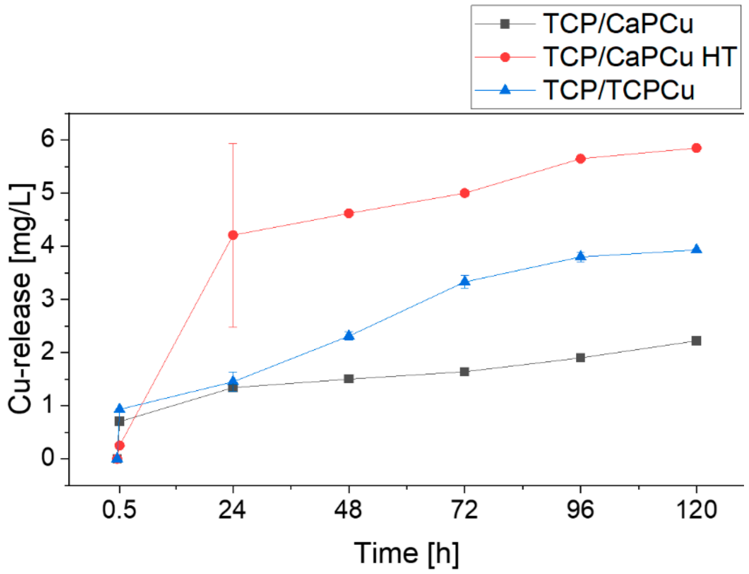

Figure S2. Cumulative Cu-release within 120 h [mg/L].

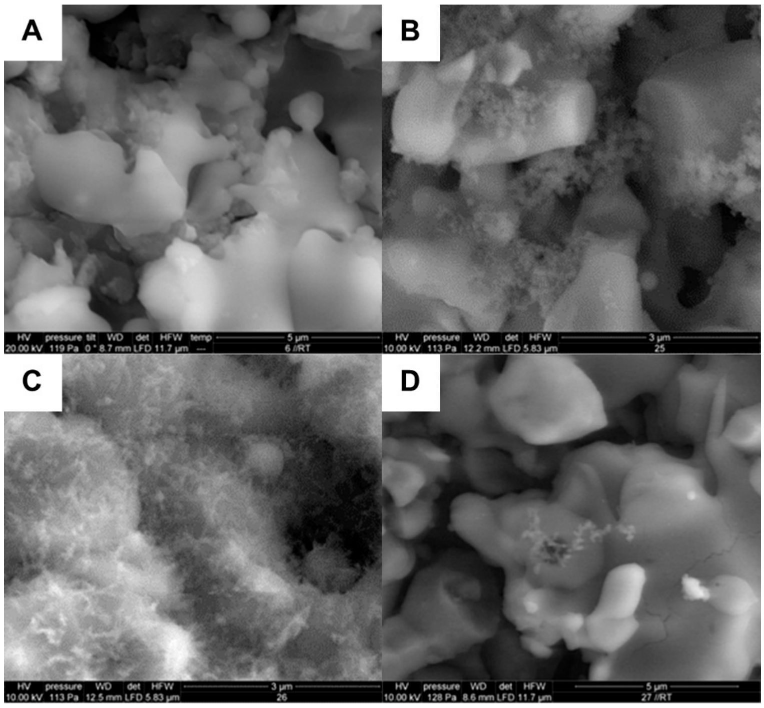

Figure S3. SEM micrographs of coatings before (A) and after 14-day immersion in SBF, TCP/CaPCu (B), TCP/CaPCu HT (C), TCP/TCPCu (D).
